# Seasonal and Spatial Variations of Bulk Nitrogen Deposition and the Impacts on the Carbon Cycle in the Arid/Semiarid Grassland of Inner Mongolia, China

**DOI:** 10.1371/journal.pone.0144689

**Published:** 2015-12-22

**Authors:** Xianglan Li, Huiqiu Shi, Wenfang Xu, Wei Liu, Xiujun Wang, Longyu Hou, Fei Feng, Wenping Yuan, Linghao Li, Hua Xu

**Affiliations:** 1 State Key Laboratory of Soil and Sustainable Agriculture, Institute of Soil Science, Chinese Academy of Sciences, Nanjing, JiangSu, China; 2 College of Global Change and Earth System Science, Beijing Normal University, Beijing, China; 3 Joint Center for Global Change Studies, Beijing, China; 4 State Key Laboratory of Vegetation and Environmental Change, Institute of Botany, the Chinese Academy of Sciences, Beijing, China; 5 University of Chinese Academy of Sciences, Beijing, China; 6 State Key Laboratory of Cryospheric Sciences, Cold and Arid Regions Environmental and Engineering Research Institute, Chinese Academy of Sciences, Lanzhou, Gansu, China; 7 Earth System Science Interdisciplinary Centre, University of Maryland, College Park, Maryland, United States of America; 8 State Key Laboratory of Earth Surface Processes and Resource Ecology, Beijing Normal University, Beijing, China; Chinese Academy of Sciences, CHINA

## Abstract

Atmospheric nitrogen (N) deposition is an important component that affects the structure and function of different terrestrial ecosystem worldwide. However, much uncertainty still remains concerning the magnitude of N deposition on grassland ecosystem in China. To study the spatial and temporal patterns of bulk N deposition, the levels of N (NH_4_^+^-N and NO_3_^-^-N) concentration in rainfall were measured at 12 sites across a 1200 km grassland transect in Inner Mongolia, China, and the respective N deposition rates were estimated. The inorganic N deposition rates ranged from 4.53 kg N ha^-1^ to 12.21 kg N ha^-1^ with a mean value of 8.07 kg N ha^-1^ during the entire growing season, decreasing steadily from the eastern to the western regions. Inorganic N deposition occurred mainly in July and August across meadow steppe, typical steppe, and desert steppe, which corresponded to the seasonal distribution of mean annual precipitation. A positive relationship was found between inorganic N deposition and mean annual precipitation (R^2^ = 0.54 ~ 0.72, *P* < 0.0001) across the grassland transect. Annual estimation of inorganic N deposition was 0.67 Pg yr^-1^ in Inner Mongolia, China based on the correlation between N deposition rates and precipitation. N deposition was an important factor controlling aboveground biomass and ecosystem respiration, but has no effect on root biomass and soil respiration. We must clarify that we used the bulk deposition samplers during the entire sampling process and estimated the dissolved NH_4_^+^-N and NO_3_^-^-N deposition rates during the entire growing season. Long-term N deposition monitoring networks should be constructed to study the patterns of N deposition and its potential effect on grassland ecosystem, considering various N species, i.e., gaseous N, particle N, and wet N deposition.

## Introduction

Nitrogen (N) deposition, an important component in the global N cycle, significantly impacts the structure and function of terrestrial ecosystems [1]. Human activities in the past few decades, including fossil fuel combustion, fertilizer production, cultivation, and urban development, have led to substantial increases in atmospheric N deposition [2]. The global rate of anthropogenic reactive N production have increased from approximately 15 Tg N yr^-1^ before 1860 to 187 Tg yr^-1^ in 2005, and is expected to double over the next 25 years [3–6]. In China, inorganic N bulk deposition increased approximately 25%, from 11.11 kg ha^-1^ yr^-1^ in the 1990s to 13.87 in the 2000s [7]. Approximately 60% of reactive N was removed from the atmosphere to the terrestrial and aquatic ecosystems via N deposition [3]. As a key limiting nutrient reactive N deposition can stimulate plant growth in N-limited regions and cause substantial CO_2_ uptake in terrestrial ecosystems if the load is not too high [8]. However, excessive atmospheric N deposition has negative impacts on terrestrial ecosystems, such as a loss of biodiversity and soil acidification [9,10]. Therefore, excess N deposition has become an important public concern due to its close relationship with human health, biodiversity, and climate change.

During the past two decades, high rates of N deposition have been widely reported in Europe [11], Africa[12], and North America [13,14]. In China, increasing N deposition and the ecological impacts of this deposition have been a great concern since the 1980s [1,7,9,15–20]. Based on current N deposition monitoring networks and published data, the total amount of N deposition was estimated to be up to 12~18 Tg N yr^-1^ in China, a relatively low value but equal to approximately 60% of the national N fertilizer consumption [18,21]. Combining site-level monitoring, gridded precipitation data, and atmospheric transport modeling results, it was estimated that N bulk deposition had increased by 59%, from 13 kg N ha^-1^ yr^-1^ in the 1960s to 20 kg N ha^-1^ yr^-1^ in the recent decade [16]. Based on ten sites observation across urban, suburban, industrial, agricultural, and rural areas during three-year period, the magnitude of total wet and dry deposition of atmospheric N species in Northern China was 60 kg N ha^-1^ with a range from 28.5 to 100.4 kg N ha^-1^ yr^-1^ because of the high rates of wet deposition and gaseous NH_3_ dry deposition [10]. Bulk N deposition in the North China Plain, an intensive agricultural region undergoing rapid economic development in China, was estimated to be 27 kg N ha^-1^ ranging from 15 to 50 kg N ha^-1^ yr^-1^[22]. Although several N deposition monitoring programs and N deposition simulation experiments have been conducted since the late 1990s [23,24], much remains unknown concerning the magnitude on different ecosystems across China because of a scarcity of measurements and quantitative knowledge [18,19]. Dissolved inorganic N deposition from eight typical forest ecosystems along the North-South Transect of Eastern China was studied, which showed that N deposition increased from north to south along the transect with a range from 1.3 to 29.5 kg ha^-1^ yr^-1^[25]. Ammonium dominated N deposition in forest ecosystem with a mean NH_4_^+^-N:NO_3_^-^-N ratio of 2.5 in bulk deposition and throughfall, and high N deposition, especially of ammonium, exceeded the critical N loads for large areas of China’s forests[26]. However, little information is available on the N deposition in grassland ecosystems. Therefore, it is crucial to study the spatial and temporal patterns of N deposition and its potential ecological impacts on grassland ecosystems and to utilize this environmentally derived nutrient resource to realize the sustainable development of grassland ecosystems.

Grassland covers approximately 40% of the land in China. Major grasslands include temperate grasslands in arid and semi-arid regions, alpine grasslands on the Tibetan Plateau, and small areas of grasslands in the warm temperate and tropical regions. Semiarid grasslands on the Mongolian Plateau are important part of the widely distributed Eurasian Steppe, including meadow steppe, typical steppe, and desert type. The seasonal and spatial variations of N deposition and its ecological effects on plant-soil-microbe interactions have been reported in typical steppe in this region [27–30]. However, the vast area and wide distribution of different types of Chinese grasslands have been largely ignored in terms of global N deposition. The objectives of this study were (1) to identify the magnitude and spatio-temporal variability of atmospheric inorganic N deposition, (2) to reveal the controlling factor on N deposition and estimate the annual N deposition in Inner Mongolia, China, (3) to analyze the ecological impacts of N deposition on the carbon cycle in grassland ecosystems.

## Methods and Materials

### 2.1 Site description

**Fig 1:**
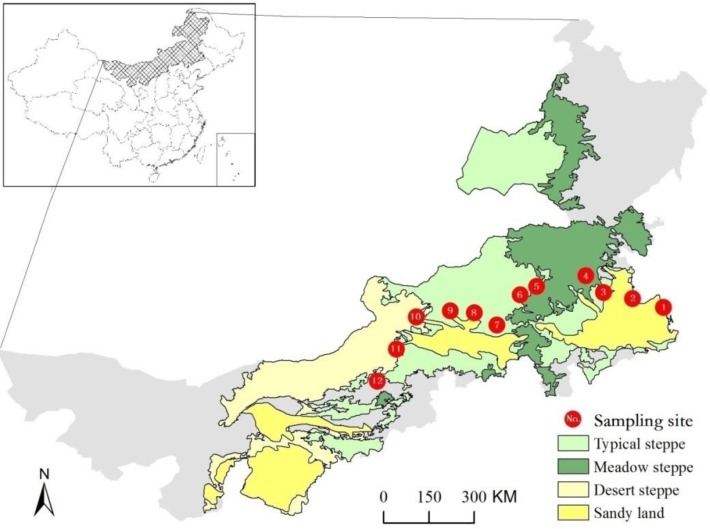
The location of the twelve sampling sites across different steppe types in Inner Mongolia, China.

Twelve monitoring sites were established to determine atmospheric N deposition, primarily the deposition of inorganic N, along a transect from Baokang (123.25 E, 44.11 N) to the Siziwang Banner (111.89 E, 41.79 N), approximately 1200 km across Inner Mongolia, China (Fig 1). Three different vegetation types, including meadow steppe (Experiment sites #1–4), typical steppe (Experiment sites #5–8), and desert steppe (Experiment sites #9–12), were included along this transect, and the distance between sites was approximately 100 km. Experiment sites #7 is a long term experimental station of Inner Mongolia grassland ecosystem operated by Chinese Academy of Sciences. Experiment sites #12 is another long term experimental station of grassland ecosystem in Siziwang Banner. The rest sites are private land rented from local farmers who gave the permission to conduct the study on these sites. All the fields studies did not involve endagered or protected species. At each site, an area of 30 m × 30 m of flat ground and homogeneous vegetation was fenced for our investigation. This transect covered a mean annual precipitation (MAP) gradient from 120 to 450 mm and a mean annual temperature gradient from 0.5 to 7.1°C. Rainfall showed a strong gradient decreasing from east to west along this transect.

### 2.2 Sampling and Measurement

#### N deposition rate

To analyze the N content in rainwater, precipitation collectors distributed evenly at all monitoring sites were installed. Four duplicate rainfall samples (bulk deposition) were collected at each site, using a glass funnel (diameter 7.5 cm) and brown bottle that was put into a PVC cube to try to prevent dust pollution. The volume of each rainfall sample was determined using a measuring cylinder. The sample was then transferred into a white plastic bottle and put into a thermostatic chamber to measure the inorganic N deposition. All of the precipitation collectors were cleaned with deionized water immediately after each collection. We collected the bulk precipitation samples included soluble and insoluble particulates every ten days during the entire grass growing season.

The unfiltered rainwater samples were frozen in a refrigerator until analysis for ammonium (NH_4_^+^-N) and nitrate (NO_3_^-^-N) by a Continuous Flow Analyzer (TRACCS2000, BranLuebbe Inc., Germany). All of the samples were collected at a frequency of 10 days and taken back to the lab as soon as possible before the dissolved inorganic nitrogen analysis. Each sample was filtered by gravity through a 0.45 μm membrane filter to remove insoluble particulates, and 15 mL filtrates were then frozen and stored in plastic bottles until chemical analysis. The methods of duplicates, blank, and standard materials were used to control data quality. NH_4_^+^-N and NO_3_^-^-N concentrations and N deposition were calculated using the following equation:

$$C=\sum_{i}^{n}Ci\times Li\;/\;\sum_{i}^{n}Li$$ (1)

Where *C* refers to the volume-weighted concentration of NH_4_^+^-N and NO_3_^-^-N (mg N L^-1^); *Ci* is the concentration of NH_4_^+^-N and NO_3_^-^-N (mg N L^-1^) for the individual event with a precipitation amount *Li* (L); and *n* refers to the number of samples taken during the entire growing season.

The deposition flux of NH_4_^+^-N and NO_3_^-^-N was calculated using the N concentrations and the amount of precipitation for each event by the following equation:

$$\text{N deposition flux}\;\left({\text{kg N ha}}^{-1}\right)=((\sum \;Ci\times 10^{-6}\times Vi)\;/\;A)\times 10000$$ (2)

Where *V*_*i*_ is the actual volume of the deposition that was collected in the brown bottle in the monitoring sites every ten days each month, and *A* the area of the glass funnel (m^2^). Nitrogen deposition per month was the sum of N deposition per event in a month. All statistical analysis was performed using SPSS 16.0 (SPSS, Inc. 2008).

#### ANPP and BNPP measurement

Aboveground net primary productivity (ANPP) and belowground net primary productivity (BNPP) were considered when evaluating the ecological impacts of N deposition on plant growth in the grassland ecosystem. To determine the ANPP, we established 3 plots of 1 m length and 1 m width which evenly distributed across each site. All plants were hand clipped at ground level. Most dead material remained attached to the grass and the ground litter content was little compared with the attached dead materials. All plants were fresh weighed, bagged and subsequently oven dried at 70°C to constant weight, and weighted to the nearest 0.1 g. For the BNPP sampling, coring was done with a root auger (8 cm inner diameter) at the depth of 0–10 cm, 10–20 cm, and 20–30 cm. Three duplications of soil samples were collected in different soil depths at each site. Once collected, root samples were brought to the laboratory where they were gently washed in a standardized sieve (2 mm) to separate roots from soil particles. Roots were then oven-dried to a constant weight at 65°C, approx. 2–3 days for the fine roots.

#### Re and Rs measurement

Ecosystem carbon exchange including ecosystem respiration (Re) and soil respiration (Rs) were considered when evaluating N deposition on carbon exchange. Static chamber-gas chromatography method was used to measure Re and Rs at an interval 10 days during the experimental period. Three static chamber were set up at each site to measure Re and Rs, respectively [31]. We clipped the grass 1–2 days before sampling to detect Rs, while no clipping was carried out to detect Re. Four gas samples from a chamber were regularly collected at an interval of 10 min between 08:00 am and 11:30 am on every sampling day. The chamber size was 50 cm×50 cm×25 cm. The CO_2_ concentration was measured by gas chromatography (Hewlett-Packard 5890 Series II). The CO_2_ fluxes were calculated according to the following equation:

$$F=\rho \times \frac{V}{A}\times \frac{\Delta c}{\Delta t}\times \frac{273}{273+T}$$ (3)

Where F stands for CO_2_ flux in mg CO_2_-C m^-2^ h^-1^, ρfor density of CO_2_ in the standard state, V for effective volume of the chamber (m^3^), A for area of the patch of field, from which CO_2_ was emitted into the chamber (m^2^), △c/△t for rate of accumulation in ppmv CO_2_-C h^-1^, and T for temperature in celsius in the chamber. Mean CO_2_ flux during the growing period was the average of the fluxes in the triplicates of each treatment weighted by the interval of two measurements.

#### Estimation of annual N deposition

To determine the annual N deposition, monthly precipitation was collected from twelve meteorological stations in Inner Mongolia that were close to the sampling sites. According to the relationship between precipitation and N deposition rates, monthly N deposition was determined. Annual N deposition was the sum of monthly N deposition in a year.

### 2.3 Data calculation and analysis

Sample differences of inorganic N deposition among sampling sites and different months were tested with the two-way analysis of variance. Comparisons of means were conducted using the Tukey’s HSD test. Regression analysis was used to examine the relation between precipitation and inorganic N deposition. Correlation between N deposition rates and precipitation was calculated during the entire grass growing season from the beginning of May to the end of September. Annual precipitation at the 12 experimental sites was taken from the stations close to these sites. Total N deposition was estimated based on the relationship formed during the entire growing season. Analysis of variance was carried out using SPSS 16.0 (SPSS, Inc. 2008) software considering the main effect of month, site, and steppe type on N deposition.

## Results

### 3.1 Seasonal variations of inorganic N deposition

**Fig 2:**
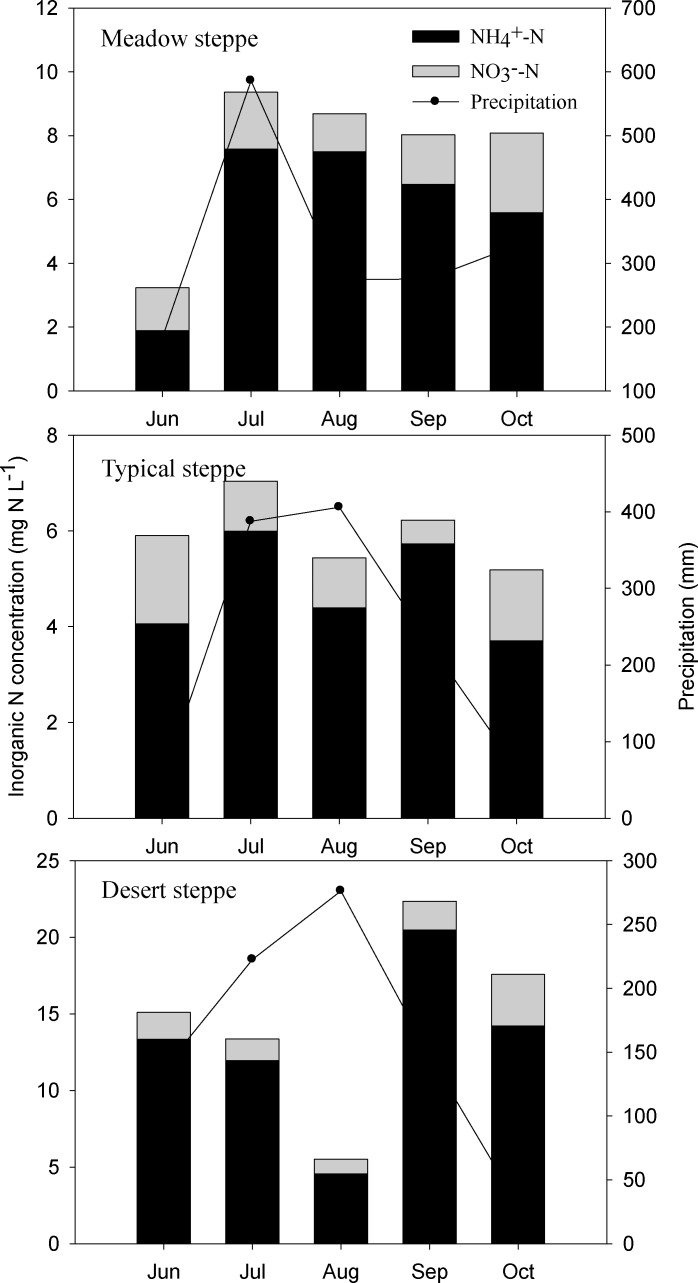
Seasonal variations of inorganic N concentrations in three different steppe types, i.e., meadow steppe, typical steppe, and desert steppe across the grassland transect during the entire growing season.

Atmospheric NH_4_^+^-N concentration was relatively higher than that of NO_3_^-^-N from three different steppe types, i.e., meadow steppe, typical steppe, and desert steppe during the entire growing season (Fig 2). Inorganic N concentration including NH_4_^+^-N and NO_3_^-^-N ranged from 1.2 to 6.4 mg N L^-1^ across the grassland transect, with a mean value of 3.1 mg N L^-1^. Concentrations of NH_4_^+^-N and NO_3_^-^-N at desert steppe sites were higher than that at the sites of meadow and typical sites by a factor of 2.1~4.1. Relatively lower inorganic N concentration was found in August in typical and desert steppe, while higher values occurred in June, July, September, and October. In meadow steppe, no significant difference of NH_4_^+^-N and NO_3_^-^-N concentration between months was found during the period from July to October.

**Fig 3:**
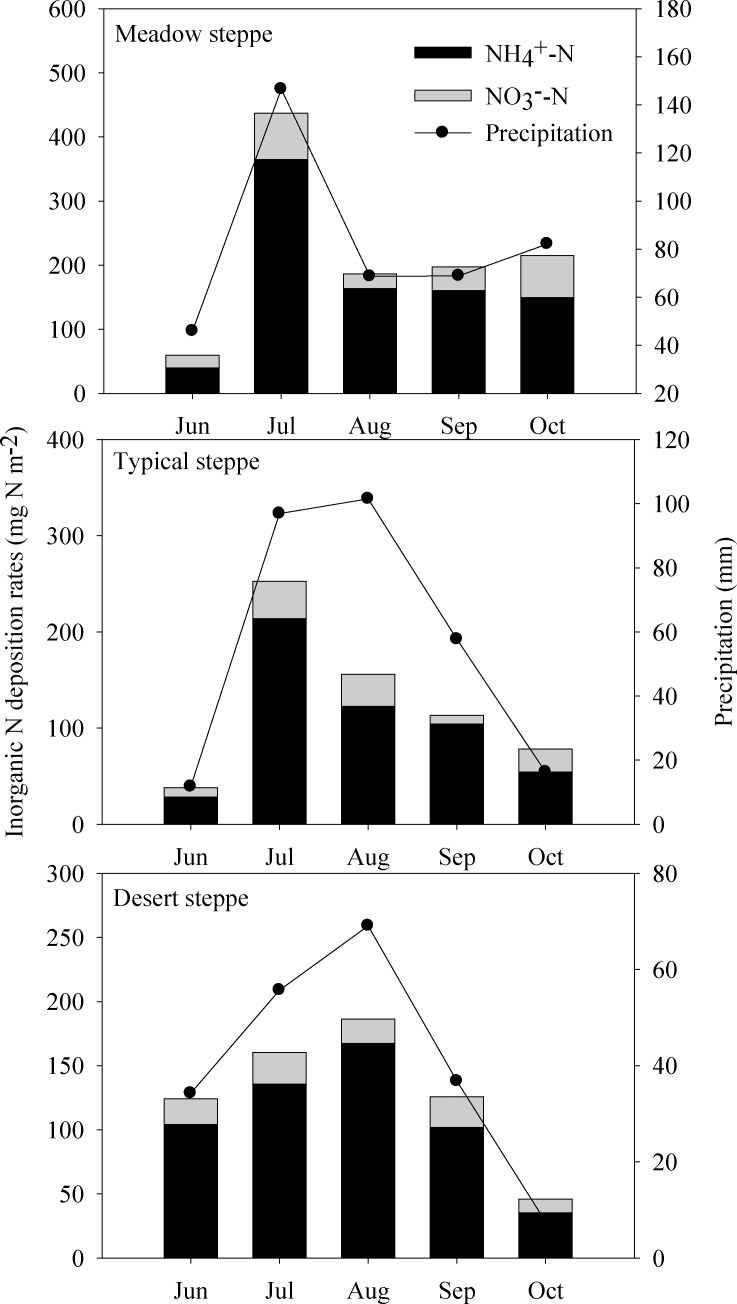
Seasonal variations of inorganic N deposition rates in three different steppes, i.e., meadow steppe, typical steppe, and desert steppe across the grassland transect during the entire growing season.

Seasonal patterns of inorganic N deposition rates (NH_4_^+^-N, NO_3_^-^-N, and NH_4_^+^-N plus NO_3_^-^-N) fluctuated dramatically during the grass growing season, with peaks occurred in July and August across different steppe types (Fig 3). High rainfall amounts occurred in July and August, accounting for 50~64% of the precipitation during the entire growing season. Overall, the deposition of inorganic N corresponded to the seasonal distribution of precipitation in Inner Mongolia, China. The monthly deposition flux of N ranged from 59 to 437 mg N m^-2^ in meadow steppe, 38 to 311 mg N m^-2^ in typical steppe, and 46 to 186 mg N m^-2^ in desert steppe, respectively (Fig 3).

Monthly mean N deposition across different steppe types ranged from 74 mg N m^-2^ in June and 309 mg N m^-2^ in July (Table 1). Statistical analysis indicated that month was an important factor controlling N deposition across the 12 experimental sites (*P* < 0.0001). Mean inorganic N deposition rates are 219, 147, and 125 mg N m^-2^ for meadow steppe, typical steppe, and desert steppe, respectively (Table 1). N deposition at the meadow steppe sites was significantly higher than those at other sites (*P* = 0.01), but similar N deposition was found between typical steppe and desert steppe (Table 1). No significant interactive effect between steppe type and month was found in this study (data not shown). This might be because N deposition rates were largely determined by precipitation pattern in different months, and there is no causal relationship between steppe type and N deposition.

**Table 1:** Inorganic N deposition rates from three different steppe types during the entire growing season in 2012. Different letters in the column of mean N deposition indicated the significance (*P* < 0.05).

Factors	Analysis of variance		
	Mean N deposition (mg N m^-2^)	F	P
Month		9.94	< 0.0001
June	74 a		
July	309 b		
August	156 c		
September	169 c		
October	110 ac		
Steppe type		4.99	0.01
Meadow steppe	219 a		
Typical steppe	147 b		
Desert steppe	125 b		

### 3.2 Spatial patterns of inorganic N deposition

**Fig 4:**
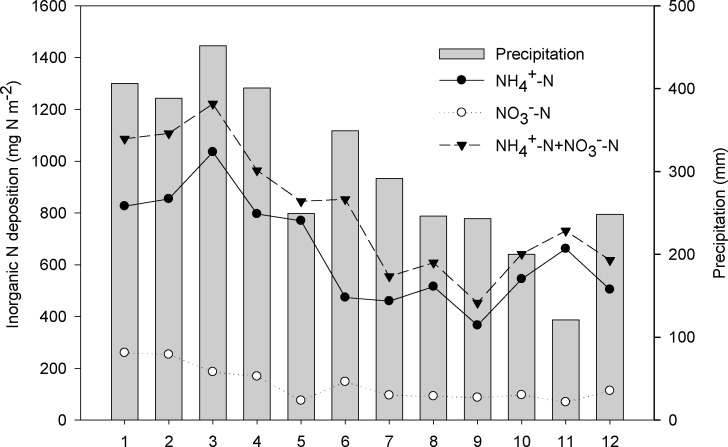
Spatial variations of the atmospheric deposition flux of N species at the 12 experimental sites.

The spatial patterns of atmospheric N deposition rates (NH_4_^+^-N, NO_3_^-^-N, and NH_4_^+^-N plus NO_3_^-^-N) along the grassland transect decreased steadily from the eastern meadow steppe to the western desert steppe regions (Fig 4). The inorganic N deposition rate ranged from 453 mg N m^-2^ (equals to 4.53 kg N ha^-1^)at Site 9 of the desert steppe to 1221 mg N m^-2^ (equals to 12.21 kg N ha^-1^) at Site 3 of the meadow steppe, with a mean value of 807 mg N m^-2^ (equals to 8.07 kg N ha^-1^) across the grassland transect. Such differences in N deposition were found across all the sites that indicated the presence of geographic trends in the spatial distribution of N deposition.

**Fig 5:**
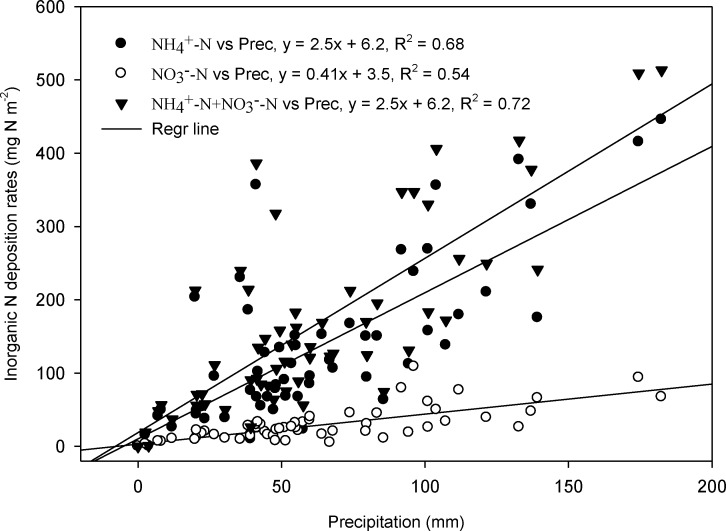
Relationship between inorganic N (NH_4_^+^-N, NO_3_^-^-N, and NH_4_^+^-N plus NO_3_^-^-N) wet deposition and precipitation at the 12 experimental sites.

Mean annual precipitation decreased from the eastern to the western sites with a range from 121 mm in desert steppe to 452 mm in meadow steppe, a trend that agreed well with the N deposition trends (Fig 4). Statistical analysis indicated that significant correlations between N deposition (NH_4_^+^-N, NO_3_^-^-N, and NH_4_^+^-N plus NO_3_^-^-N) and precipitation were found across all the sites (R^2^ = 0.54~ 0.72), showing that precipitation was an important factor controlling the geographic patterns in inorganic N deposition in Inner Mongolia, China (Fig 5).

### 3.3 ANPP and BNPP

Both monthly and mean ANPP decreased regularly from the eastern to western sites and reached the lowest values at desert steppe (Table 2; S1 Fig), following the similar tendency with precipitation (Fig 4). Overall, aboveground biomass decreased with the decreasing N deposition rate (R^2^ = 0.40, *P* < 0.05) and precipitation (R^2^ = 0.60, *P* < 0.0001) (Table 2; S1 Fig).

**Table 2:** Plant growth and ecosystem carbon exchange at 12 sites across the grassland transect in Inner Mongolia, China.

Sites	ANPP (kg ha^-1^)	BNPP (kg ha^-1^)			Re (mg m^-2^ h^-1^)	Rs (mg m^-2^ h^-1^)
		0–10 cm	10–20 cm	20–30 cm		
1	12.7	25.1	5.86	4.25	599	358
2	8.56	16.3	5.92	3.60	754	417
3	7.70	26.6	6.68	4.39	440	181
4	8.60	21.0	6.04	4.10	705	333
5	6.61	35.7	18.6	10.4	425	274
6	5.74	18.1	18.1	12.0	515	374
7	5.23	24.5	14.1	7.67	373	212
8	5.38	23.4	11.8	6.92	485	246
9	4.56	23.0	7.06	4.62	265	188
10	5.34	15.7	6.88	5.67	289	194
11	1.68	9.31	5.30	2.82	126	91
12	7.46	22.7	8.83	6.08	390	196

Both monthly and mean BNPP from different soil depths (0~10 cm, 10~20 cm, and 20~30 cm) showed different spatial patterns across the grassland transect, showing no significant relationship between BNPP and inorganic N deposition and precipitation (Table 2; S2 Fig).

### 3.4 Re and Rs

Both Re and Rs decreased regularly from the eastern to western sites and reached the lowest values at desert steppe of site #11 (Table 2). Significant positive relationship was found between precipitation and ecosystem respiration (R^2^ = 0.64, *P* < 0.05) and soil respiration (R^2^ = 0.44, *P* < 0.05) (S3 Fig). However, no significant correlation was found between N deposition and Re and Rs (R^2^ = 0.24~0.39, *P>* 0.05) (S3 Fig).

## Discussion

### 4.1 Factors controlling N deposition rates across the grassland transect

Both natural factors and human activities are important for controlling N deposition rates across different land surface ecosystems. Across the grassland transect, precipitation at the eastern sites showed higher values than that at the western sites. However, the opposite trend was observed for the inorganic N concentration in rainwater. It indicated that significant positive relationships between bulk N deposition rates and precipitation (Fig 5). Previous studies have reported consistent effects of precipitation on the N deposition [22,32], suggesting that the amount of rainfall influenced the seasonal trends of N deposition at a given site.

**Fig 6:**
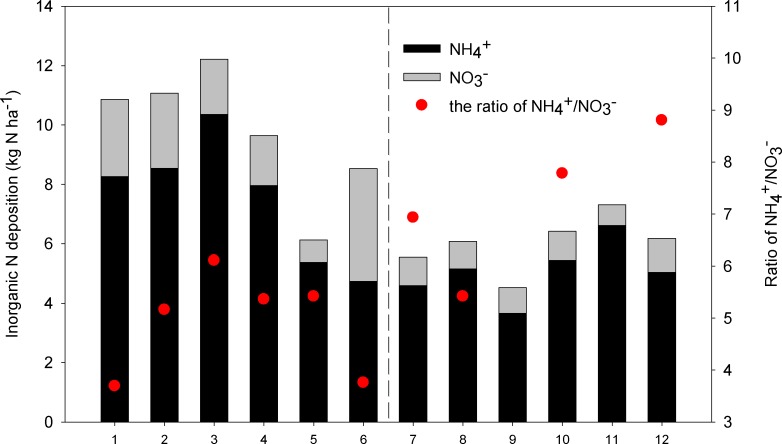
Dissolved inorganic N deposition and the ratio of NH_4_^+^-N/NO_3_^-^-N during the entire growing season at the 12 experimental sites.

The main anthropogenic source of NH_4_^+^-N in the atmosphere was generally considered to be NH_3_ volatizing from N fertilizers in agricultural and natural areas and from the excrements of human beings and animals, while major anthropogenic sources of NO_3_^-^-N were NOx emitted from fossil fuel combustion in industries, vehicles and biomass burning [10]. Hence, the ratio of NH_4_^+^-N/NO_3_^-^-N may indicate the relative contribution of reactive N from agriculture and animal husbandry and from industry and traffic emissions to N deposition on the local/regional scale. Determining these relative contributions of reactive N may allow for additional source information to be obtained concerning N deposition [33]. In this study, the NH_4_^+^-N deposition rate was, on average, 5.3 times greater than that of NO_3_^-^-N, with a range from 3.2 to 10.2 times greater than that of NO_3_^-^-N across different sites of the transect (Fig 6). This difference was more pronounced at the western sites, thereby indicating that NH_4_^+^-N was a greater contributor to inorganic N in the desert steppe than in the meadow steppe and typical steppe. Furthermore, NH_4_^+^-N from grassland ecosystems and animal excrement might be remained the major contributor to N deposition in the desert steppe area, compared with NO_3_^-^-N from fossil fuel combustion in the industrial and transportation sectors in the meadow steppe regions, where human activities were much more frequent than in the other sites. Therefore, in addition to precipitation, human activity was another controlling factor that affected the spatial patterns of N deposition along the grass transect in Inner Mongolia, China.

### 4.2 Estimation of annual N deposition in grassland ecosystem in Inner Mongolia

**Fig 7:**
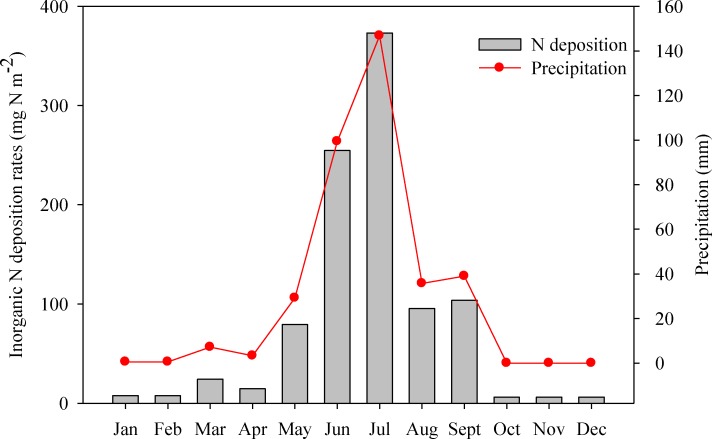
Monthly variations of precipitation at meteorological stations and N estimation in Inner Mongolia, China.

According to the relationship between precipitation and N deposition (NH_4_^+^-N plus NO_3_^-^-N) at different sites across the grassland transect, annual N deposition was estimated according to the monthly precipitation collected from the meteorological stations (Fig 7). Annual N deposition was 979 mg N m^-2^ (equals to 9.79 kg N ha^-1^), with high peaks occurred during the period from May to September, accounting for 93% of the annual deposition rates. Accordingly, measured N deposition was 8.07 kg N ha^-1^ during the entire growing season, accounting for 82% of the estimated value. The available area of grassland in Inner Mongolia was 6.8×10^11^ m^2^ in total, and the N deposition was estimated to be 0.67 Pg yr^-1^ in this study.

In this study, the deposition of inorganic N ranged from 4.53 to 12.21 kg N ha^-1^ yr^-1^ with a mean value of 8.07 kg N ha^-1^ yr^-1^ during the period from June to October across the grassland transect in Inner Mongolia (Fig 6), were relatively low compared to the overall values and those reported for other areas in China [10,18]. The average wet deposition fluxes of inorganic N were 13.87 kg N ha^-1^ yr^-1^ in China and 25 kg N ha^-1^ yr^-1^ in North China [7]. Considering dissolved organic N in wet deposition and NH_3_, HNO_3_, and particulates NH_4_^+^-N plus NO_3_^-^-N in dry deposition, the average wet plus dry deposition of N was 60.6 kg N ha^-1^ yr^-1^ over agricultural and industrial regions in Northern China during the period from 2008 to 2010 [10].

The relatively low deposition of inorganic N may be explained by three factors. First, the observed period was only 5 months long, spanning the entire growing season, but not a whole year. However, this might only play a minor role since the N deposition estimation in May to September accounted for 93% of the annual N deposition (Fig 7). Second, the underlying surface in our study was grassland and did not include agriculture, industry, and urban areas. Although human activities especially industry development increased NO_3_^-^-N and decreased the ratio of NH_4_^+^-N and NO_3_^-^-N in eastern region of Inner Mongolia, it could not alter local N deposition rates completely. This is because the NO_3_^-^-N deposition rates were relatively low when compared with NH_4_^+^-N deposition (Fig 4). Third, inorganic N species NH_4_^+^-N and NO_3_^-^-N were considered in this study. Dissolved organic N comprises approximately 30% of total N deposition[34], but organic N in deposition was not considered in this study. N species of NH_3_, HNO_3_, and particulates NH_4_^+^-N and NO_3_^-^-N in dry deposition were not taken into account. The thematic difference such as inorganic, organic, dry N deposition might be the primary reason for the differences we have observed. Annual deposition of inorganic N was 47 mmol m^-2^ yr^-1^; 51% of atmospheric deposition was attributed by dry deposition [38]. The dry and N deposition should be estimated to be about 16 kg N ha^-1^ which was comparable with the previous studies [13,14,38]. Dry N deposition should be involved in the future study.

### 4.3 Ecological impacts of inorganic N deposition

The increase in atmospheric N deposition can alter rates of C and N cycles of terrestrial ecosystems, and thus affect the structure and function of terrestrial ecosystems. Over the last decade, numerous simulated N deposition experiments have been conducted in different ecosystems in China [1,9,17]. Previous studies have focused on the potential effects of atmospheric N deposition on grassland ecosystem, including aboveground productivity, biodiversity, acidification, the potential of soil C storage and fixation, and the emission of greenhouse gases [9,21]. ANPP, BNPP, Re, and Rs were considered when evaluating impacts of N deposition on carbon cycle in grassland ecosystem (Table 2).

Previous studies found that N was a major limiting factor in the growth of grasslands since N addition may lessen the N limitation by increasing soil N availability and thus stimulate plant growth [29,35]. However, previous studies also indicated that ANPP of arid and semiarid ecosystems was primarily limited by the temporal and spatial patterns of precipitation, and the effect of N on ANPP was usually neglected [36,37]. A four-year study with several N application levels executed in Inner Mongolia grasslands indicated that N produced no effect on ANPP under dry conditions, however, ANPP can be improved by four times the mean value in this region with over 105 kg N ha^-1^ application under relative conditions. This means that if water restrictions are eased, N becomes another important limiting factor [27].

BNPP was a major source of organic C pool in the soil, with more than 60% of annual C originating from plants. Changes in biomass allocation patterns may impact C and N fluctuations, corresponding to resource availability and management practices. Previous studies indicated that In North and Central American grasslands, root biomass increases along with N availability [38]. Oppositely, results from an alpine meadow in India exhibited a decrease of root biomass after two-year’s N application [39]. Therefore, belowground vegetation biomass was controlled by comprehensive factors including soil N availability and water content.

Previous reports indicated diverse effects of N input on CO_2_ effluxes in grassland ecosystems. For example, increased N-inputs have shown to retard mineralization of soil organic matter and depress soil respiration CO_2_ losses [40]. N deposition tended to decrease CO_2_ emission in an alpine meadow on the Qinghai-Tibetan Plateau, but all the differences caused by N deposition were all not significant [41]. However, other researchers have observed small increases in soil respiration in response to increased N-inputs [42]. Nitrogen addition tended to promote soil CO_2_ effluxes and this effect increased with the N addition levels; the decrease in soil CO_2_ efflux from low N addition was mainly attributed to decrease of aboveground biomass [43]. Previous study found that CO_2_ effluxes remained unchanged upon five years of chronic N addition, and these authors suggested that abiotic mechanisms might play a great role in N retention [35].

Two mechanisms needed to be considered when thinking about the effect of N deposition on soil respiration and ecosystem respiration. First, N deposition increases plant growth, which increases uptake of CO_2_ and the rate of carbon sequestration [44]. Also, N additions can suppress rates of soil decomposition and release of CO_2_ to the atmosphere. Scarcity of plant-available N often limits plant growth rates, such that N additions typically enhance rates of net primary production (CO_2_ uptake) across various terrestrial ecosystems, including temperate and tropical forests, tundra, grasslands, and wetlands [35]. Second, the reduced soil respiration in response to increased N deposition might be caused by a combination of reduced root respiration when N becomes more available and reduced microbial demand for recalcitrant forms of N-containing organic matter [40,45].

### 4.4 The limitation of the sampling technique

We must clarify that bulk rather than wet deposition was collected in this study. The wet deposition referred strictly to wet-only deposition which was collected only during rainfall and snowfall events since the samplers were closed outside the periods of precipitation[20]. The bulk deposition referred to rainfall and snowfall samples collected using traditional rain gauges which were open permanently[20]. This suggested that bulk deposition contained wet plus unquantifiable dry deposition (including N species of both gases and particles) and therefore it should be higher than wet deposition [22]. For the rainwater collectors in this study, a glass funnel inserted into a brown bottle and put into a PVC cube, was used in this study. Although some insects and particles were prevented to come into the brown bottle, bulk deposition such as particles and gaseous N was in fact monitored using this equipment. In general, N deposition sampled by bulk sampler was higher than that of wet-only device but lower than that combined from individual sampling technique [46]. Thus the method used in this study might underestimate the total N deposition [47]. It is critically important to consider various N species (NH_x_ and NO_y_, particles, and wet N deposition) to quantify both the wet and the dry deposition; otherwise, an extrapolation of the total N deposition flux could yield a high underestimation.

## Conclusions

Bulk precipitation samples were collected to study the dissolved inorganic nitrogen in grassland ecosystem along a 1200 km transect in Inner Mongolia, China. Inorganic N deposition rates decreased steadily from the east to the west along the grassland transect, exhibiting significant spatial and temporal patterns. Ammonium dominated N deposition in grassland ecosystem with a mean NH_4_^+^-N:NO_3_^-^-N ratio of 5.3 in bulk deposition. NH_4_^+^-N/NO_3_^-^-N ratios in N deposition clearly increased from the eastern to the western regions, which was most likely caused by the increase in NO_3_^-^-N from fossil fuel consumption and the rapid growth of industry and transportation at the eastern sites. Both precipitation and anthropogenic activities played an important role in defining the spatial and temporal patterns of atmospheric N deposition. Significant impacts of N deposition on aboveground biomass and ecosystem respiration was found. Different N species in bulk precipitation such as dissolved organic N, particles and gaseous N should be considered in the future studies. The patterns of the total N deposition and its potential effects on grassland ecosystem should be identified through long-term N deposition monitoring networks and cross-site N addition experiments in China.

## Supporting Information

S1 Fig Spatial changes of monthly ANPP and the correlation between ANPP and precipitation and N deposition across the 12 monitoring sites in Inner Mongolia, China. (DOCX)

S2 Fig Spatial changes of monthly BNPP at different soil depths and the correlation between BNPP and precipitation and N deposition across the 12 monitoring sites in Inner Mongolia, China. (DOCX)

S3 Fig Spatial changes of ecosystem respiration (Re) and soil respiration (Rs) and the correlation between carbon exchange and precipitation and N deposition at the 12 monitoring sites in Inner Mongolia, China. (DOCX)
