# Supplementary material for: Seasonal and Spatial Variations of Bulk Nitrogen Deposition and the Impacts on the Carbon Cycle in the Arid/Semiarid Grassland of Inner Mongolia, China
Source: PLoS One. 2015 Dec 22;10(12):e0144689. doi: 10.1371/journal.pone.0144689 (PMC4687917; doi:10.1371/journal.pone.0144689)
Supplement: S1 Fig — (DOCX) [file pone.0144689.s001.docx]

SUPPORTING INFORMATION 1:

S1 Figure Legend: Spatial changes of monthly ANPP and the correlation between ANPP and precipitation and N deposition across the 12 monitoring sites in Inner Mongolia, China.


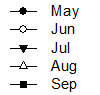


R^2^=0.40, *P* ＜ 0.05

R^2^=0.60, *P* ＜ 0.0001
