# Supplementary material for: Seasonal and Spatial Variations of Bulk Nitrogen Deposition and the Impacts on the Carbon Cycle in the Arid/Semiarid Grassland of Inner Mongolia, China
Source: PLoS One. 2015 Dec 22;10(12):e0144689. doi: 10.1371/journal.pone.0144689 (PMC4687917; doi:10.1371/journal.pone.0144689)

SUPPORTING INFORMATION 2:

S2 Figure Legend: Spatial changes of monthly BNPP at different soil depths and the correlation between BNPP and precipitation and N deposition across the 12 monitoring sites in Inner Mongolia, China.


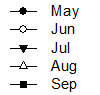

Supplement: S2 Fig — (DOCX) [file pone.0144689.s002.docx]
