# Supplementary material for: Seasonal and Spatial Variations of Bulk Nitrogen Deposition and the Impacts on the Carbon Cycle in the Arid/Semiarid Grassland of Inner Mongolia, China
Source: PLoS One. 2015 Dec 22;10(12):e0144689. doi: 10.1371/journal.pone.0144689 (PMC4687917; doi:10.1371/journal.pone.0144689)
Supplement: S3 Fig — (DOCX) [file pone.0144689.s003.docx]

SUPPORTING INFORMATION 3:

S3 Figure Legend: Spatial changes of ecosystem respiration (Re) and soil respiration (Rs) and the correlation between carbon exchange and precipitation and N deposition at the 12 monitoring sites in Inner Mongolia, China.


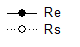


R^2^=0.24～0.39

*P＞* 0.05

R^2^=0.44～0.64

*P* ＜ 0.05
